# Planning a Clinical Practice Guideline Revision for a Core Set of Outcome Measures in Adults Undergoing Neurologic Physical Therapy, Part 2: Determining Stakeholders' Uptake and Recommendations

**DOI:** 10.1002/gin2.70085

**Published:** 2026-09-06

**Authors:** Jane E. Sullivan, Kirsten Potter, Marghuretta Bland, Elizabeth Anderl, Wendy Romney, Zeynep Gulen Erkoc, Jennifer L. Moore

**Affiliations:** ^1^ Department of Physical Therapy and Human Movement Sciences, Feinberg School of Medicine Northwestern University; ^2^ Tufts University – School of Medicine, Department of Rehabilitation Sciences, Doctor of Physical Therapy Seattle; ^3^ Washington University School of Medicine, Program in Physical Therapy; ^4^ TIRR Memorial Hermann; ^5^ Department of Physical Therapy and Human Movement Science Sacred Heart University; ^6^ Groton School Groton MA; ^7^ Institute of Knowledge Translation, and Regional Rehabilitation Knowledge Center, Sunnaas Rehabilitation Hospital Oslo Norway

**Keywords:** clinical practice guideline, neurology, outcome measures, physical therapy

## Abstract

**Objective:**

To determine the uptake and recommendations for revision of the 2018 Clinical Practice Guideline (CPG): Core Set Outcome Measures for Adults with Neurologic Conditions Undergoing Rehabilitation.

**Design:**

Online Survey.

**Setting:**

Online with participants from multiple settings.

**Participants:**

Licensed physical therapists (PT) or physical therapist assistants (PTA) or a non‐PT supervisor of PTs and/or PTAs.

**Interventions:**

N/A.

**Main Outcome Measures:**

An online survey was developed to determine respondents' knowledge, perceived benefits, challenges, gaps, and modifications made to the 2018 CPG as well as to ascertain respondents' recommendations for the CPG revision. Data were analyzed descriptively by respondent role (clinician, educator/researcher, or supervisor/manager). Respondents' narrative comments were coded into 3 categories: implementation challenges, modifications, and revision recommendations.

**Results:**

Four hundred and ninety‐seven individuals accessed the survey. Seventy‐six percent (379) completed 80% of items. Two hundred and forty‐one (63.5%) identified their primary role as clinician. The majority (99%) agreed they understood the purpose, process and recommendations of the CPG; 70% agreed that the CPG changed their practice. More than 70% agreed the CPG‐recommended Core Set provided a comprehensive assessment of balance and gait. Narrative comments revealed implementation challenges including lack of time, space and buy‐in. Clinicians made modifications to the Core Set including using fewer measures, adding other measures and modifying measures (e.g. changing distances, providing patient assistance). Suggestions for the CPG revision included adding higher and lower‐level measures.

**Conclusions:**

Despite reported barriers to clinical implementation of CPGs, survey respondents overwhelmingly reported gaining knowledge and changing practice in response to the 2018 CPG. They recommended the revision include measures to capture both higher‐ and lower‐level activities. Future CPG developers should consider assessing uptake and recommendations to help guide CPG revisions.

## Summary

- To inform the update to a 2018 CPG on the use of outcome measures in neurological physical therapist practice, an online survey was used to determine respondents' knowledge, perceived benefits, challenges, gaps, and modifications made when using the 2018 CPG recommendations in practice.
- Survey respondents were asked to provide their recommendations for the CPG update.
- Survey data showed that the majority of respondents understood the purpose, process, and recommendations of the 2018 CPG and agreed that it improved their knowledge and changed their practice.
- We recommend that future CPG developers consider assessing uptake and recommendations to help guide CPG revisions.

## 1 Introduction

A clinical practice guideline (CPG) is a collection of action statements based on the systematic review of literature on a focused topic that makes recommendations on clinical practice intended to optimize patient care, minimize harm, and reduce unnecessary variability [1, 2, 3]. CPGs are an outcome of one of the objectives of the American Physical Therapy Association (APTA) strategic plan [4]. *A Core Set of Outcome Measures for Adults with Neurologic Conditions Undergoing Rehabilitation: A CLINICAL PRACTICE GUIDELINE* was published in 2018 [5]. The CPG was co‐sponsored by the APTA and the Academy of Neurologic Physical Therapy (ANPT). The focus of the CPG was informed by two stakeholder surveys: a clinician survey completed by 303 physical therapists and physical therapist assistants and a consumer survey completed by 215 individuals with a neurologic condition who had received physical therapy. The results from those surveys are briefly summarized in the CPG [5]. Analysis of survey responses led the guideline development group to focus on outcome measures (OMs) that had acceptable clinical utility; could be used to assess change in a client's balance, gait, transfers, and patient‐stated goals; and could be used for adults with neurologic conditions across practice settings [5].

Experts recommend regular revisions of CPGs; however, there is no consensus on the appropriate timeframe for revisions to occur. Most concur that CPG revisions are appropriate when the literature or practice changes [6, 7, 8, 9]. Surveys are a common tool used to inform decision‐making to update CPGs. In the ANPT, a 2016 CPG addressed the management of those with unilateral peripheral vestibular hypofunction and was revised in 2022 [10]. The Academy of Pediatric Physical Therapy published a CPG on the management of congenital muscular torticollis in 2013 [11]. Four years later, the CPG authors undertook a survey to explore the uptake of that guideline [12]. Information gathered via the survey helped inform the revised guideline that was published the following year [13]. However, although the Academy of Orthopedic Physical Therapy has published several CPG revisions which outline the revision process, it does not appear that the CPG developers attempted to ascertain uptake or public input on the revisions which is typically done [14, 15, 16, 17]. While strategies for assessing CPG uptake and priorities for revision vary [9], prior to a CPG revision, it seems prudent to examine the impact of the existing CPG to help set priorities for the revision.

While CPGs provide a foundation for evidence‐based care, research consistently shows that they are often underutilized in clinical settings. Despite clinicians' awareness of, and agreement with guideline recommendations, research suggests that they are not followed approximately two‐thirds of the time [18]. This breakdown in the publication–dissemination–implementation pipeline suggests that patients may not fully benefit from advances in research. In rehabilitation, several studies echo these concerns. Al Zoubi and colleagues conducted a systematic review of implementation strategies aimed at increasing uptake of musculoskeletal CPGs and identified 11 studies using multifaceted approaches to improve physical therapists' use of recommendations, with effects ranging from small to large [19]. However, four of those studies did not improve patient outcomes. Kaplan and colleagues examined the implementation of the congenital muscular torticollis CPG in pediatric settings and found that although uptake improved with targeted dissemination and implementation efforts, the effectiveness of those strategies was limited [12]. Similarly, Salbach et al. conducted a large cluster randomized trial across 20 rehabilitation sites to implement 18 stroke rehabilitation recommendations and found no significant improvement when comparing active implementation strategies to passive dissemination [20]. These findings illustrate that without deliberate efforts to assess and facilitate uptake, even well‐designed guidelines may have limited impact on practice, underscoring the need to systematically study CPG uptake as part of the revision process.

The purpose of this study was to examine the uptake of the 2018 CPG (initial CPG) in clinical practice, education, and research, and to inform the update of the CPG (update CPG). The impact of those data on the proposed CPG revision will be discussed.

## 2 Methods

The purposes of the revision survey were to determine CPG uptake utility and to query respondents about priorities for a revision. To build a survey to inform the revision, authors re‐visited the surveys done prior to the initial CPG. Domains were added to explore how the initial CPG has been used in the clinic, in education and in research; gaps and challenges and if/how these have been addressed; as well as priorities for the CPG update. Items were grouped by respondent type (clinician, researcher/educator and manager/supervisor) so that respondents answered only questions relevant to their primary role, totaling 70–90 items. The survey included multiple sections: demographics/respondent characteristics; knowledge and perceived benefit of the CPG; clinical utility, including challenges to use and modifications made; and priorities for the revision. Item responses were yes/no, 5‐point Likert Scale (strongly agree—strongly disagree), and open‐text responses. A copy of the survey can be seen in Supporting Figure 1.

### 2.1 Data Collection

The survey was administered online using Qualtrics and was available for 6 months from February—July 2024. Invitations to participate were distributed via email listservs and e‐newsletters from the ANPT, QR codes at a national APTA conference and via the researchers' personal social media feeds (e.g., LinkedIn, Facebook). The ANPT is a 6000 member organization [21]. The APTA has over 100,000 members [22]. The inclusion criteria to participate in this study included: (1) being a licensed physical therapist (PT) or physical therapist assistant (PTA) or a non‐PT supervisor of PTs and/or PTAs (2) having provided informed consent. Respondents were asked to indicate that they met these criteria before beginning the survey.

### 2.2 Data Analysis

Quantitative survey data were analyzed to determine the frequency and percentage of responses for each item. Several survey items allowed respondents to provide open text responses. These included items about challenges, modifications made when using the Core Set measures, and gaps that should be addressed in the CPG update. Open text response items were analyzed using a quantitative (descriptive) content analysis that combined deductive and inductive elements. While survey items were designed around domains (e.g. challenges and recommendations), responses were pooled and analyzed collectively because of the conceptual overlap of responses across items. For example, a respondent may have described a suggestion for the CPG revision in their response to the item about gaps or modifications in the CPG. The reviewers (WR and JES) independently reviewed all open text items and inductively generated an initial set of codes. Through an iterative process, codes were refined and organized into four overarching categories: challenges, modifications, gaps, and suggestions. Although these categories broadly reflect the survey domains, they were derived from patterns observed from the data rather than assigned a priori. A coding framework, including category definitions and associated codes, was created through consensus. Each open text response was then coded using this framework. Responses could be assigned multiple codes and categories when applicable. A third author (ZGE) audited the coding, referred any discrepancies back to the initial coders, and ultimately all coding decisions were unanimous across the three reviewers. Frequencies of codes and categories were calculated to summarize response patterns.

## 3 Results

*Respondents:* A total of 497 individuals accessed the survey. Seventy‐six percent (379) completed at least 80% of items. This included 63.5% (241) who identified their primary role as clinicians; 27.2% (103) educator/researchers; 5.8% (22) manager/supervisors; and 3.7% (14) as others. Most respondents were American Board of Physical Therapy Specialities Board‐certified Neurologic Clinical Specialists (clinicians 61%; educator/researchers 70%; supervisors/managers 59%). Overwhelmingly, respondents reported belonging to the APTA and ANPT (> 98% and > 85%, respectively). Table 1 provides data on survey respondents by role.

**Table 1:** Respondent demographics.

Clinicians *n* = 241			
Work setting		PT/PTA staff in facility	
Acute care	4.4%	< 5	**5%**
IRF	30.6%	5–10	28%
SNF	0%	11–20	26%
LTAC	0.3%	> 20	40%
Long‐term NH	0%	**Years practicing**	
OP (hospital‐based)	31.2%	0–4	16%
OP (private)	4.1%	5–9	35.5%
Home Health	1.6%	10–14	20%
Non‐response	27.7%	15–19	9%

**Educator/researchers *n*** = **103**			
**Years practicing**		**Years teaching**	
0–4	1.9%	0–4	26.2%
5–9	27.2%	5–9	27.2%
10–14	18.4%	10–14	20.4%
15–19	9.7%	15–19	2%
> 20	16.5%	> 20	0%

**Supervisor/Managers *n*** = **22**			
**Work setting**		**PT/PTA Staff in Facility**	
Inpatient rehabilitation	36.4%		
Outpatient (private)	31.8%	< 5	4.5%
Home health	13.6%	5–10	3.4%
Skilled nursing facility	4.5%	11–20	9.1%
Acute care (AC)	4.5%	> 20	50%
Long‐term AC	0%		
Long‐term nursing facility	0%		
Outpatient (hospital‐based)	0%		
Other	0%		
Years of experience			
0–4	41%		
5–9	41%		
10–14	4.5%		
15–19	4.5%		
> 20	10%		

*Note:* percentages are based on the actual number of respondents completing the item.

Abbreviations: AC‐acute care; IRF‐inpatient rehabilitation facility; LTAC‐long term acute care facility; NH‐nursing home; OP‐ outpatient; PT‐physical therapist; PTA‐physical therapist assistant; SNF‐skilled nursing facility.

### 3.1 Knowledge, Benefits, and Impact of the CPG

Most respondents (99%) understood the “purpose, process, and recommendations of the Core Set of Outcome Measures CPG.” Nearly half of respondents strongly agreed that the CPG changed their knowledge (clinicians = 52.28%; educators/researchers = 47.57%; supervisors/managers 50%), and more than 70% strongly agreed that the CPG changed their practice (work). These data suggest strong uptake of the CPG recommendations.

#### 3.1.1 Clinical Utility

Participants were questioned about the importance of clinical utility factors such as time to administer measures, cost of measure, and necessary equipment to perform the measure. Across all roles, more than half of respondents indicated that administration time and cost were “very important” to whether a measure is used in the clinic. However, only 48% of educators/researchers and less than 15% of clinicians and managers viewed equipment as “very important.” See Table 2 for details.

**Table 2:** Clinical utility and core set use responses.

	Respondents' answers to item, “For each clinical utility factor below, identify its importance in selecting core set measures.”		
	Administration time	Cost	Equipment
Clinicians *n* = 241			
Very important	85.89%	65.15%	8.30%
Somewhat important	10.37%	30.71%	1.66%
Not important	2.90%	2.49%	2.49%
Educator/researchers *n* = 2103			
Very important	82.52%	62.14%	48.15%
Somewhat important	15.53%	35.92%	7.41%
Not important	1.94%	1.94%	14.81%
Supervisor/managers *n* = 22			
Very important	50.00%	63.64%	12.62%
Somewhat important	27.27%	36.36%	1.94%
Not important	9.09%	0.00%	3.88%
Administrative questions (asked only of supervisor/managers)			
Are there fields in the electronic health record for the Core Set measures?			
Berg balance scale	91%		
Functional gait assessment	86%		
10‐Meter walk test	86%		
6‐min walk test	86%		
Activities‐specific balance confidence scale	65%		
None	5%		
Other than in clinical care, for what other purposes does your organization use data from the Core Set?			
Quality improvement (QI) initiatives	76%		
Reporting at family/team conferences	71%		
Reporting to payers	57%		
Staff evaluation and development	52%		
Appealing billing‐related denials	48%		
Marketing	14%		
Comparison of outcomes across organizations	14%		

*Note:* Percentages are based on the actual number of respondents completing the item.

#### 3.1.2 Comprehensiveness of Core Set Measures

Several survey items were designed to ascertain if the CPG provided a comprehensive measure of two primary target constructs, balance and gait. More than 70% of the respondents agreed or strongly agreed that the Core Set provided a comprehensive measure of balance (clinicians 89%; educator/researchers 71%; managers/supervisors 78%) and gait (clinicians 92%; educator/researchers 86%; managers/supervisors 100%).

#### 3.1.3 Core Set Use in Clinical Practice

Slightly less than half of clinician and supervisor/manager respondents reported that their facilities had a required Core Set of outcome measures (Clinicians – 46%, supervisors/managers – 45%). The most frequently included facility‐required measures from the initial CPG were the Berg Balance Scale, 6‐min and 10‐Meter Walk Test. Least frequently included was the Activities‐Specific Balance Confidence Scale.

#### 3.1.4 Core Set Use in Clinical Administration

Supervisors/managers were asked about whether their electronic health record had fields to capture Core Set scores. They were also asked about non‐clinical uses of the Core Set data. Their responses can be found in Table 2.

#### 3.1.5 Measure Use, Standardization and Scoring

Sixty‐eight percent of managers/supervisors reported that measures are standardized in their clinics. More than 90% of responding clinicians said they use the ANPT standardization procedures for test administration. Over half of the educator/researchers (51.46%) and nearly half of clinicians (48.9%) “strongly agreed” a test should be administered even though a patient might score a 0.

#### 3.1.6 Core Set Use in Education

The majority (97%) of educator respondents reported teaching their students to administer all recommended Core Set outcome measures. Most said students were taught to use the Core Set at initial exam (100%), discharge (96%) and at least one time in between (75%). Few (17.5%) said that their students were required to read the CPG. A small percent (10%) had used the standardized administration protocols developed by the Core Measures CPG Knowledge Translation task force in their teaching.

#### 3.1.7 Challenges, Modifications, and Gaps in the CPG

One hundred and eighty comments described challenges respondents experienced when using the Core Set. The most frequently identified challenges included time to administer (*n* = 43) and appropriateness to patient population (*n* = 42). In addition, space requirements and lack of standardization issues were described by clinicians (*n* = 9).

Clinicians and manager/supervisors made 143 comments about multiple Core Set modifications, including using fewer measures (*n* = 51), modifying Core Set measures (*n* = 35), and using additional measures (*n* = 22).

#### 3.1.8 Suggestions for CPG Revision

Eighty‐eight comments for suggestions were made regarding the CPG revision. Clinician and educator/researcher respondents recommended that the Core Set revision consider adding higher‐level (clinicians *n* = 18/educator/research *n* = 5) and lower‐level (clinicians *n* = 13/educator/researcher *n* = 3) measures. Table 3 lists the coding of open text responses by category and role.

**Table 3:** Coding of open text responses.

Coding category	Number of open text responses that were coded		
	Clinicians	Educator/researchers	Manager/supervisors
Challenges related to use of the core set − time to administer − equipment − cost	146	25	9
Modifications made to core set − replace measures − reduce measure − added measures − deleted ABC	131	*	12
Gaps in the core set − lower level measures − higher level measures	146	31	15
Suggestions for CPG revision − add cognitive and arm function measures	55	24	9

*Survey item not asked of those in this role.

## 4 Discussion

### 4.1 Uptake

A primary goal of the survey was to assess uptake of the recommendations from the initial 2018 CPG. Respondents' data confirmed that the CPG influenced knowledge and practice for physical therapists in clinical, educational/research and supervisory roles. This is consistent with a recent study that reported a significant increase in use (exceeding 70%) in the 1 year after implementing a CPG‐recommended measurement battery [23]. Further, this study reported that physical therapists surveyed indicated that implementation of the measurement battery resulted in positive changes [23]. The positive impact on practice aligns with ANPT's 2018–2022 strategic plan to translate evidence into practice, ensure high‐value care, inform future research, and improve communication between ANPT and providers to ensure access to education, networking and key resources [24]. Current revision efforts remain consistent with the ANPT strategic plan to advance neurologic physical therapy through knowledge creation and translation through the development of quality resources [25].

Most survey respondents felt that using the Core Set provided a comprehensive examination of two target constructs, balance and gait. A recent study concluded that implementation of a CPG‐recommended measurement battery focused on balance and gait was both feasible and sustainable for 22 physical therapists working in an inpatient rehabilitation facility [23]. Multiple additional benefits to Core Set use were identified, including staff development, quality improvement (QI) initiatives, and communication with patients/clients, their families, and payors. Despite the multiple additional benefits listed, it was surprising that only 14% used data for marketing. This limited use contrasts with evidence that systematic outcome tracking can strengthen organizational visibility, demonstrate value to stakeholders, and support sustainability of evidence‐based practices [26, 27, 28]. Since organizations are using the Core Set for QI and knowledge translation (KT) initiatives this may represent lost opportunities to maximize the potential of the Core Set data for organizational marketing in practice. Future KT efforts could target broader application/use of the OM data.

Many educators report teaching Doctor of Physical Therapy (DPT) students to administer the Core Set measures. Educators report they teach students to use the CPG recommendations for measure administration frequency (at initial, interim, and discharge examinations). Although most respondents support measure standardization, only 10% of educators reported using the administration protocols developed by the CPG KT taskforce in their teaching. This gap between awareness and standardized use mirrors other findings in physical therapy research, where variability in assessment training can undermine measurement fidelity and reliability [29, 30, 31, 32]. This may present a future opportunity to develop skilled clinicians who use standardized administration procedures to enhance the reliability of repeated measures in the clinic. Similarly, these protocols can provide valid examination data to help practitioners when making clinical decisions.

Time and cost of examinations were rated as key considerations in measure selection by respondents. They reported experiencing implementation challenges such as space and time, and some chose to modify Core Set measures, delete some measures, or add measures. Our findings align with Kaplan and colleagues' survey on the uptake of the CPG on treatment of Congenital Muscular Torticollis [12]. In addition to reporting implementation data, their respondents identified barriers including time and resistance to change. Less than half of our respondents reported that their facility requires use of the Core Set measures yet many respondents reported that there are fields in the EMR for the Core Set measures. Administrative support of recommended standardized measure use is key to facilitate adoption.

Surprisingly, the ABC, a self‐reported measure, was used least frequently as compared to the five other Core Set performance‐based measures. Our respondents chose self‐reported measures more frequently in outpatient settings. This aligns with a 2021 recommendation to use self‐report measures to evaluate return to work, a common goal in the outpatient setting [33]. Since more than half of our respondents work in inpatient settings, we speculate the work setting may influence measure choice. These findings may also be aligned with data from a 2025 survey on the use of standardized outcome measures in physical therapy. In which respondents agreed performance‐based measures “help me make a clinical decision” and were willing to dedicate more time to perform performance‐based measures [34].

### 4.2 Revision Recommendations

There were 88 unique suggestions regarding the CPG revision. Some recommended inclusion of measures that better capture individuals with both higher and lower abilities, reflecting a desire for greater responsiveness across the continuum of neurologic recovery. The Guideline Development Group (GDG) plans to continue to target constructs (e.g., balance, gait) as search terms in the revision. This approach aligns with best‐practice recommendations that guideline updates remain focused on pre‐defined constructs while systematically evaluating new evidence for measures that address a broader range of ability [7, 9]. This means that measures that can be used to examine the spectrum of balance (high to low level) will be reviewed if the relevant literature meets inclusion criteria. Some respondents recommended including measures outside the scope of the initial CPG (e.g., cognition, arm function). Since the CPG revision will target the same constructs as the 2018 CPG (balance, gait, transfers, and patient‐stated goals), only measures that capture these constructs will be included. However, since our respondents suggested these additional areas be explored, other CPG groups may choose to include these in their scope of review.

The revision recommendations should reflect the priorities expressed by respondents. The fact that more than half of respondents saw administration time and cost as “very important” suggests that measures in the revised CPG may have stronger clinical utility if they are quick to administer and low or no cost. On the other hand, less than half of respondents viewed equipment as “very important” and this may reflect that the existing Core Set measures already prioritizes measures requiring minimal equipment. This discrepancy suggests that further reductions in time and cost may yield greater implementation gains than efforts to further minimal equipment demands.

The inclusion of additional measures as identified through the survey may inadvertently expand the intent of the Core Set, leaving clinicians with too many options. The GDG applied the COSMIN framework to establish a minimal set of recommended measures for use in clinical practice. Although COSMIN was developed for research purposes, it was adapted by the GDG for clinical practice. We believe the minimum data set should remain tailored to the realities of clinical practice. Any measure additions should be evaluated closely by the GDG based on available evidence, recognizing that expanding beyond the minimal set may further increase the time barrier for clinicians. Less than 50% of respondents reported their facility‐required use of the entire Core Set. However, facility‐required use of individual Core Set measures was rated much higher. The use of the entire Core Set was the intention of the original CPG. The GDG authors will consider individual versus the set as they discuss the purpose of the revision.

The recommendations made by respondents highlight a common challenge in guideline development of balancing the desire for comprehensive measurement with the need for feasible implementation in clinical practice. Expanding to include measures that capture a broader range of ability may enhance clinical relevance but could also increase time and resource demands, potentially limiting adoption [30, 31]. As the CPG is revised, each proposed addition will need to be evaluated not only for its psychometric strength but also for its alignment with the clinical utility criteria emphasized in the original guideline, time to administer, cost, equipment needs, and interpretability [5]. Ensuring that any new measures meet these practical benchmarks will help maintain the usability and sustainability of the Core Set in routine practice. This approach also aligns with recommendations from implementation and measurement science frameworks that emphasize feasibility and appropriateness as key determinants of successful adoption [27, 35].

### 4.3 Limitations

There are several limitations to our work. Firstly, we used a sample of convenience. Our data may not be representative of all practicing PT/PTAs. Invitations to participate in the survey were widely disseminated via professional communication and social media. While 76% of those who accessed the survey actually completed all items, an actual response rate cannot be calculated. The ANPT has approximately 6000 members [21] and APTA represents over 10,000 [22]. “Respondents were eligible to complete the survey if they agreed that they were either a licensed physical therapist (PT) or physical therapist assistant (PTA) or a non‐PT supervisor of PTs and/or PTAs.” “A similar survey on CPG uptake [12] did not report a response rate. Like our survey, this may have biased the sample towards an evidence‐based audience with different experiences from the survey sample.” Our respondents were seasoned (years of practice, specialist certification, ANPT members) with high representation from inpatient rehabilitation facilities and hospital‐based outpatient clinics and none from skilled nursing or long‐term care settings. Survey participation may have been biased towards individuals already engaged with the CPG or outcome measure initiatives. While the survey was adapted from prior instruments used in the original CPG development, modifications were not formally validated. Although open‐text responses were independently coded and consensus achieved, interpretation is inherently influenced by coder perspective and may not fully capture the respondents' intent. Although survey items were grouped by primary role, many respondents function across multiple roles (e.g., clinician‐educator), which may have influenced response patterns. Findings reflect perspectives at a single point in time, approximately 7 years after guideline publication, and may not represent current or future implementation contexts. We were unable to assess change in practice since the initial CPG survey did not examine actual practice; rather, we sought to define the CPG scope. Finally, while consumers were surveyed prior to the initial CPG, that was not done after the CPG was published.

## 5 Conclusions

Despite reported barriers to CPG uptake in clinical practice, our data confirm that implementation of the 2018 Core Set CPG has been successful. Although CPG revision timeframes and processes are not standardized [10, 13], we urge future CPG revision groups to investigate stakeholder uptake and recommendations before planning their work.

## Author Contributions

**Jane E. Sullivan:** conceptualization, investigation, funding acquisition, writing – original draft, methodology, validation, visualization, writing – review and editing, formal analysis, project administration, resources, software, data curation, supervision. **Kirsten Potter:** conceptualization, investigation, funding acquisition, writing – original draft, writing – review and editing, visualization, validation, methodology, software, formal analysis, project administration, data curation, supervision, resources. **Marghuretta Bland:** conceptualization, investigation, funding acquisition, writing – original draft, writing – review and editing, visualization, validation, methodology, software, formal analysis, project administration, resources, supervision, data curation. **Elizabeth Anderl:** conceptualization, investigation, funding acquisition, writing – original draft, methodology, validation, visualization, writing – review and editing, project administration, formal analysis, software, data curation, supervision, resources. **Wendy Romney:** resources, supervision, data curation, software, formal analysis, project administration, writing – review and editing, visualization, validation, methodology, conceptualization, investigation, funding acquisition, writing – original draft. **Zeynep Gulen Erkoc:** software, writing – review and editing, investigation. **Jennifer L. Moore:** conceptualization, investigation, funding acquisition, writing – original draft, writing – review and editing, visualization, validation, methodology, software, formal analysis, project administration, resources, supervision, data curation.

## Funding

The authors have nothing to report.

## Ethics Statement

This survey was approved by Tufts University IRB#00004522.

## Conflicts of Interest

Drs. Sullivan, Moore, and Potter were co‐authors of the 2018 CPG.

## Supporting information


Supporting File

## Data Availability

Data sharing not applicable to this article as no datasets were generated or analyzed during the current study.

## References

[1] R. M. Rosenfeld and R. N. Shiffman , “Clinical Practice Guidelines: A Manual for Developing Evidence‐Based Guidelines to Facilitate Performance Measurement and Quality Improvement,” supplement, Otolaryngology–Head and Neck Surgery: Official Journal of American Academy of Otolaryngology‐Head and Neck Surgery 135, no. 4 Suppl (2006): 1–28, 10.1016/s0194-5998(06)03094-4. 17023260

[2] Institute of Medicine Committee on Standards for Developing Trustworthy Clinical Practice G ., Clinical Practice Guidelines We Can Trust, eds. R. Graham , M. Mancher , D. Miller Wolman , S. Greenfield , and E. Steinberg (National Academies Press (US) Copyright 2011 by the National Academy of Sciences. All rights reserved, 2011). 24983061

[3] S. L. Kaplan , C. Coulter , and L. Fetters , “Developing Evidence‐based Physical Therapy Clinical Practice Guidelines,” Pediatric Physical Therapy 25, no. 3 (2013): 257–270, 10.1097/PEP.0b013e31829491c5. 23743574

[4] Association APT , “APTA Strategic Plan 2022‐2025 Alexandria, VA.2022,” [cited 2024 3‐2‐24], https://www.apta.org/contentassets/14be4b91c1b94da8a92dfb76fe041b99/apta-strategic-plan-2022-2025.pdf.

[5] J. L. Moore , K. Potter , K. Blankshain , S. L. Kaplan , L. C. O'Dwyer , and J. E. Sullivan , “A Core Set of Outcome Measures for Adults With Neurologic Conditions Undergoing Rehabilitation: A Clinical Practice Guideline,” Journal of Neurologic Physical Therapy 42, no. 3 (2018): 174–220, 10.1097/npt.0000000000000229. PMC6023606 29901487

[6] M. H. Murad , “Clinical Practice Guidelines,” Mayo Clinic Proceedings 92, no. 3 (2017): 423–433, 10.1016/j.mayocp.2017.01.001. 28259229

[7] L. Martínez García , I. Arévalo‐Rodríguez , I. Solà , R. B. Haynes , P. O. Vandvik , and P. Alonso‐Coello , “Strategies for Monitoring and Updating Clinical Practice Guidelines: A Systematic Review,” Implementation Science 7 (2012): 109, 10.1186/1748-5908-7-109. PMC3520818 23164220

[8] P. G. Shekelle , E. Ortiz , S. Rhodes , et al., “Validity of the Agency for Healthcare Research and Quality Clinical Practice Guidelines: How Quickly Do Guidelines Become Outdated?,” Journal of the American Medical Association 286, no. 12 (2001): 1461–1467, 10.1001/jama.286.12.1461. 11572738

[9] R. W. Vernooij , A. J. Sanabria , I. Solà , P. Alonso‐Coello , and L. Martínez García , “Guidance for Updating Clinical Practice Guidelines: A Systematic Review of Methodological Handbooks,” Implementation Science 9 (2014): 3, 10.1186/1748-5908-9-3. PMC3904688 24383701

[10] C. D. Hall , S. J. Herdman , S. L. Whitney , et al., “Vestibular Rehabilitation for Peripheral Vestibular Hypofunction: An Updated Clinical Practice Guideline From the Academy of Neurologic Physical Therapy of the American Physical Therapy Association,” Journal of Neurologic Physical Therapy 46, no. 2 (2022): 118–177, 10.1097/npt.0000000000000382. PMC8920012 34864777

[11] S. L. Kaplan , C. Coulter , and L. Fetters , “Physical Therapy Management of Congenital Muscular Torticollis: An Evidence‐Based Clinical Practice Guideline: From the Section on Pediatrics of the American Physical Therapy Association,” Pediatric Physical Therapy: The Official Publication of the Section on Pediatrics of the American Physical Therapy Association 25, no. 4 (2013): 348–394, 10.1097/PEP.0b013e3182a778d2. 24076627

[12] S. L. Kaplan , R. L. Dole , and J. Schreiber , “Uptake of the Congenital Muscular Torticollis Clinical Practice Guideline Into Pediatric Practice,” Pediatric Physical Therapy 29, no. 4 (2017): 307–313, 10.1097/pep.0000000000000444. 28953172

[13] S. L. Kaplan , C. Coulter , and B. Sargent , “Physical Therapy Management of Congenital Muscular Torticollis: A 2018 Evidence‐Based Clinical Practice Guideline From the APTA Academy of Pediatric Physical Therapy,” Pediatric Physical Therapy 30, no. 4 (2018): 240–290, 10.1097/pep.0000000000000544. PMC8568067 30277962

[14] K. R. Enseki , N. J. Bloom , M. Harris‐Hayes , et al., “Hip Pain and Movement Dysfunction Associated With Nonarthritic Hip Joint Pain: A Revision,” Journal of Orthopaedic & Sports Physical Therapy 53, no. 7 (2023): CPG1–CPG70, 10.2519/jospt.2023.0302. 37383013

[15] A. J. H. Arundale , M. Bizzini , C. Dix , et al., “Exercise‐Based Knee and Anterior Cruciate Ligament Injury Prevention,” Journal of Orthopaedic & Sports Physical Therapy 53, no. 1 (2023): CPG1–CPG34, 10.2519/jospt.2023.0301. 36587265

[16] S. Z. George , J. M. Fritz , S. P. Silfies , et al., “Interventions for the Management of Acute and Chronic Low Back Pain: Revision 2021,” Journal of Orthopaedic & Sports Physical Therapy 51, no. 11 (2021): CPG1–CPG60, 10.2519/jospt.2021.0304. PMC10508241 34719942

[17] R. L. Martin , T. E. Davenport , J. J. Fraser , et al., “Ankle Stability and Movement Coordination Impairments: Lateral Ankle Ligament Sprains Revision 2021,” Journal of Orthopaedic & Sports Physical Therapy 51, no. 4 (2021): CPG1–CPG80, 10.2519/jospt.2021.0302. 33789434

[18] S. Mickan , A. Burls , and P. Glasziou , “Patterns of ‘Leakage’ in the Utilisation of Clinical Guidelines: A Systematic Review,” Postgraduate Medical Journal 87, no. 1032 (2011): 670–679, 10.1136/pgmj.2010.116012. PMC3181428 21715571

[19] F. M. Al Zoubi , A. Menon , N. E. Mayo , and A. E. Bussières , “The Effectiveness of Interventions Designed to Increase the Uptake of Clinical Practice Guidelines and Best Practices Among Musculoskeletal Professionals: A Systematic Review,” BMC Health Services Research 18, no. 1 (2018): 435, 10.1186/s12913-018-3253-0. PMC5994025 29884165

[20] N. M. Salbach , S. Wood‐Dauphinee , J. Desrosiers , et al., “Facilitated Interprofessional Implementation of a Physical Rehabilitation Guideline for Stroke in Inpatient Settings: Process Evaluation of a Cluster Randomized Trial,” Implementation Science 12, no. 1 (2017): 100, 10.1186/s13012-017-0631-7. PMC5539742 28764752

[21] Therapy AoNP . Membership Stats Alexandria VA: ANPT; 2025 [cited 2026 April 27,2026], https://www.neuropt.org/about-us/membership-stats.

[22] Association APT , APTA Annual Report ‐ 2025 (APTA, 2025), https://www.apta.org/siteassets/pdfs/2025/reports/apta-annual-report-2025.pdf.

[23] L. Ling , R. Carson , G. N. Olivier , and M. A. French , “Implementation of a Gait and Balance Battery of Outcome Measures in an Inpatient Rehabilitation Hospital,” Physical Therapy 106 (2026), 10.1093/ptj/pzag059. PMC13403179 42213082

[24] Therapy AoNP , ANPT Strategic Plan 2018‐2022 (ANPT, 2018), https://www.neuropt.org/docs/default-source/about/strategic-plan/anpt_strategic_plan_2020-update.pdf?sfvrsn=29985043_0.

[25] Therapy AoNP , Strategy Mountain 2023‐2026 (ANPT, 2023), https://www.neuropt.org/docs/default-source/members-only-docs/anpt-2024-strategy-mountain.pdf?sfvrsn=2e955b43_1.

[26] E. Suter , N. Oelke , C. Adair , and G. Armitage , “Ten Key Principles for Successful Health Systems Integration,” Healthcare Quarterly 13 Spec No, no. Spec No (2009): 16–23, 10.12927/hcq.2009.21092. PMC3004930 20057244

[27] E. Proctor , H. Silmere , R. Raghavan , et al., “Outcomes for Implementation Research: Conceptual Distinctions, Measurement Challenges, and Research Agenda,” Administration and Policy in Mental Health and Mental Health Services Research 38, no. 2 (2011): 65–76, 10.1007/s10488-010-0319-7. PMC3068522 20957426

[28] L. J. Damschroder , D. C. Aron , R. E. Keith , S. R. Kirsh , J. A. Alexander , and J. C. Lowery , “Fostering Implementation of Health Services Research Findings into Practice: A Consolidated Framework for Advancing Implementation Science,” Implementation Science 4 (2009): 50, 10.1186/1748-5908-4-50. PMC2736161 19664226

[29] N. Salbach , S. Guilcher , and S. Jaglal , “Physical Therapists' Perceptions and Use of Standardized Assessments of Walking Ability Post‐Stroke,” Journal of Rehabilitation Medicine 43, no. 6 (2011): 543–549, 10.2340/16501977-0820. 21533335

[30] D. U. Jette , J. Halbert , C. Iverson , E. Miceli , and P. Shah , “Use of Standardized Outcome Measures in Physical Therapist Practice: Perceptions and Applications,” Physical Therapy 89, no. 2 (2009): 125–135, 10.2522/ptj.20080234. 19074618

[31] R. A. Swinkels , R. P. van Peppen , H. Wittink , J. W. Custers , and A. J. Beurskens , “Current Use and Barriers and Facilitators for Implementation of Standardised Measures in Physical Therapy in the Netherlands,” BMC Musculoskeletal Disorders 12 (2011): 106, 10.1186/1471-2474-12-106. PMC3126762 21600045

[32] D. Riese , J. Kool , J. Sieben , and R. de Bie , “Education of Physiotherapists Improves Inter‐Rater Reliability in Lumbar Spine Motor Control Tests: A Randomized Controlled Trial,” Musculoskeletal Science & Practice 64 (2023): 102741, 10.1016/j.msksp.2023.102741. 36878140

[33] D. Daley , L. P. Payne , J. Galper , et al., “Clinical Guidance to Optimize Work Participation After Injury or Illness: The Role of Physical Therapists,” Journal of Orthopaedic & Sports Physical Therapy 51, no. 8 (2021): CPG1–CPG102, 10.2519/jospt.2021.0303. 34338006

[34] A. Kristal , I. A. Gaunaurd , S. J. Morgan , et al., “Use of Standardized Outcome Measures Among Physical Therapists in the United States: A Cross‐Sectional Survey Study,” PLoS One 20, no. 8 (2025): e0330528, 10.1371/journal.pone.0330528. PMC12367168 40834029

[35] C. A. C. Prinsen , L. B. Mokkink , L. M. Bouter , et al., “Cosmin Guideline for Systematic Reviews of Patient‐Reported Outcome Measures,” Quality of Life Research 27, no. 5 (2018): 1147–1157, 10.1007/s11136-018-1798-3. PMC5891568 29435801

