# Correction to “Planning a Clinical Practice Guideline Revision for a Core Set of Outcome Measures in Adults Undergoing Neurologic Physical Therapy, Part 2: Determining Stakeholders' Uptake and Recommendations”

**DOI:** 10.1002/gin2.70092

**Published:** 2026-10-08

J. E. Sullivan, K. Potter, M. Bland, et al., “Planning a Clinical Practice Guideline Revision for a Core Set of Outcome Measures in Adults Undergoing Neurologic Physical Therapy, Part 2: Determining Stakeholders’ Uptake and Recommendations,” *Clinical and Public Health Guidelines* 3 (2026): e70085. https://doi.org/10.1002/gin2.70085.

In Table 2, line 5, there is a typo in the header, “Educator/researchers *n* = **2**103”:

**TABLE 2**. Clinical utility and core set use responses.

**Table:** 

	Respondents' answers to item, “For each clinical utility factor below, identify its importance in selecting core set measures.”		
	Administration time	Cost	Equipment
Clinicians *n* = 241			
Very important	85.89%	65.15%	8.30%
Somewhat important	10.37%	30.71%	1.66%
Not important	2.90%	2.49%	2.49%
Educator/researchers *n* = 2103			
Very important	82.52%	62.14%	48.15%
Somewhat important	15.53%	35.92%	7.41%
Not important	1.94%	1.94%	14.81%
Supervisor/managers *n* = 22			
Very important	50.00%	63.64%	12.62%
Somewhat important	27.27%	36.36%	1.94%
Not important	9.09%	0.00%	3.88%
Administrative questions (asked only of supervisor/managers)			
Are there fields in the electronic health record for the Core Set measures?			
Berg balance scale	91%		
Functional gait assessment	86%		
10‐Meter walk test	86%		
6‐min walk test	86%		
Activities‐specific balance confidence scale	65%		
None	5%		
Other than in clinical care, for what other purposes does your organization use data from the Core Set?			
Quality improvement (QI) initiatives	76%		
Reporting at family/team conferences	71%		
Reporting to payers	57%		
Staff evaluation and development	52%		
Appealing billing‐related denials	48%		
Marketing	14%		
Comparison of outcomes across organizations	14%		

*Note:* Percentages are based on the actual number of respondents completing the item.

It should read, “Educator/researchers *n* = 103”:

**TABLE 2**. Clinical utility and core set use responses.

**Table:** 

	Respondents' answers to item, “For each clinical utility factor below, identify its importance in selecting core set measures.”		
	Administration time	Cost	Equipment
Clinicians *n* = 241			
Very important	85.89%	65.15%	8.30%
Somewhat important	10.37%	30.71%	1.66%
Not important	2.90%	2.49%	2.49%
Educator/researchers *n* = 103			
Very important	82.52%	62.14%	48.15%
Somewhat important	15.53%	35.92%	7.41%
Not important	1.94%	1.94%	14.81%
Supervisor/managers *n* = 22			
Very important	50.00%	63.64%	12.62%
Somewhat important	27.27%	36.36%	1.94%
Not important	9.09%	0.00%	3.88%
Administrative questions (asked only of supervisor/managers)			
Are there fields in the electronic health record for the Core Set measures?			
Berg balance scale	91%		
Functional gait assessment	86%		
10‐meter walk test	86%		
6‐min walk test	86%		
Activities‐specific balance confidence scale	65%		
None	5%		
Other than in clinical care, for what other purposes does your organization use data from the Core Set?			
Quality improvement (QI) initiatives	76%		
Reporting at family/team conferences	71%		
Reporting to payers	57%		
Staff evaluation and development	52%		
Appealing billing‐related denials	48%		
Marketing	14%		
Comparison of outcomes across organizations	14%		

This has now been corrected in the published article.

We apologize for this error.

