# Supplementary material for: Planning a Clinical Practice Guideline Revision for a Core Set of Outcome Measures in Adults Undergoing Neurologic Physical Therapy, Part 2: Determining Stakeholders' Uptake and Recommendations
Source: Clin Public Health Guidel. 2026 Sep 6;3(4):e70085. doi: 10.1002/gin2.70085 (PMC13618638; doi:10.1002/gin2.70085)
Supplement: Supplementary file 1 [file GIN2-3-e70085-s001.docx]

**Figure 1: Stakeholder Uptake and Recommendation Survey**

**What is your PRIMARY role? Please select the role that aligns best with the majority of your work related to measurement.**

- Clinician
- Educator/Researcher
- Manager/Supervisor/Administrator
- Other: if other, what is your PRIMARY role

Following this question, respondents were presented with survey items specific to their identified role.

**Clinician Questions**

*As you answer the questions that follow, please respond as related to your primary position as a clinician.*

**I understand the purpose, processes, and recommendations of the Core Set of Outcome Measures CPG.**

- Yes
- No

**The Core Set of Outcome Measures CPG changed my knowledge.**

- Yes, if YES HOW?
- No

**The Core Set of Outcome Measures CPG changed my work.**

- Yes, if YES HOW?
- No

**The Core Set provides a comprehensive measure of balance.**

- Strongly agree
- Agree
- Neutral
- Disagree
- Strongly disagree

**The Core Set provides a comprehensive measure of gait.**

- Strongly agree
- Agree
- Neutral
- Disagree
- Strongly disagree

**Does your facility have a required set of outcome measures? Select all that apply.**

- No
- Yes, including the Berg Balance Scale
- Yes, including the Functional Gait Assessment
- Yes, including the Activities Specific Balance Confidence (ABC) Scale
- Yes, including the 10-Meter Walk Test
- Yes, including the 6-Minute Walk Test
- Yes, including the 5 Times Sit to Stand Test
- Other: Please comment

**For patients with goals and capacity to improve static and dynamic balance, when do you use the Berg Balance Scale? Select all that apply.**

- Never
- At initial evaluation
- At discharge
- At least once in between
- For patients with acute conditions (<6 months since diagnosis)
- For patients with chronic stable conditions (>6 months since diagnosis)
- For patients with chronic progressive conditions (>6 months since diagnosis)

**For patients with goals and capacity to improve balance while walking, when do you use the Functional Gait Assessment? Select all that apply.**

- Never
- At initial evaluation
- At discharge
- At least once in between
- For patients with acute conditions (<6 months since diagnosis)
- For patients with chronic stable conditions (>6 months since diagnosis)
- For patients with chronic progressive conditions (>6 months since diagnosis)

**For patients with goals and capacity to improve balance confidence, when do you use the Activities Specific Balance Confidence (ABC) Scale? Select all that apply.**

- Never
- At initial evaluation
- At discharge
- At least once in between
- For patients with acute conditions (<6 months since diagnosis)
- For patients with chronic stable conditions (>6 months since diagnosis)
- For patients with chronic progressive conditions (>6 months since diagnosis)

**For patients with goals and capacity to improve walking speed, when do you use the 10-Meter Walk Test? Select all that apply.**

- Never
- At initial evaluation
- At discharge
- At least once in between
- For patients with acute conditions (<6 months since diagnosis)
- For patients with chronic stable conditions (>6 months since diagnosis)
- For patients with chronic progressive conditions (>6 months since diagnosis)

**For patients with goals and capacity to improve walking distance, when do you use the 6-Minute Walk Test? Select all that apply.**

- Never
- At initial evaluation
- At discharge
- At least once in between
- For patients with acute conditions (<6 months since diagnosis)
- For patients with chronic stable conditions (>6 months since diagnosis)
- For patients with chronic progressive conditions (>6 months since diagnosis)

**For patients with goals and capacity to improve walking speed, when do you use the 5-Times Sit to Stand Test? Select all that apply.**

- Never
- At initial evaluation
- At discharge
- At least once in between
- For patients with acute conditions (<6 months since diagnosis)
- For patients with chronic stable conditions (>6 months since diagnosis)
- For patients with chronic progressive conditions (>6 months since diagnosis)

**When do you examine and document a patient’s goals using an outcome measure such as the Goal Attainment Scale? Select all that apply.**

- Never
- At initial evaluation
- At discharge
- At least once in between
- For patients with acute conditions (<6 months since diagnosis)
- For patients with chronic stable conditions (>6 months since diagnosis)
- For patients with chronic progressive conditions (>6 months since diagnosis)

**I feel it is important to administer a measure in the Core Set even if a patient may score near a 0 on the test.**

- Strongly agree
- Agree
- At discharge
- Neutral
- Disagree
- Strongly disagree

**I use the Academy of Neurologic Physical therapy Core Set standardized administration procedures?** Never

- Always
- Frequently
- Seldom
- Never

**I feel confident suing results from standardized outcome measures to: select all that apply.**

- Have a conversation with a patient
- Inform discussions with payers
- Inform a patient’s prognosis
- Formulate a PT diagnosis for the patient
- Make intervention-related decisions
- Other: please comment

**When do you outcome measures with patients? Select all that apply.**

- Never
- At initial evaluation
- At discharge
- At least once in between
- For patients with acute conditions (<6 months since diagnosis)
- For patients with chronic stable conditions (>6 months since diagnosis)
- For patients with chronic progressive conditions (>6 months since diagnosis)

**For each clinical utility factor below, identify its importance in selecting Core Set measures. Please indicate any other clinical utility factors that should be used as a criterion for selecting measures.**

|  | **Very Important** | **Somewhat important** | **Not important** |
| --- | --- | --- | --- |
| Time to administer and score: < 20 minutes/ measure |  |  |  |
| Cost: available at no cost |  |  |  |
| Equipment: no specialized equipment |  |  |  |

Other: please comment:

**Have you modified the Core Set in your setting?**

- **Yes: if yes, please describe**
- **No**

**Are there any gaps you believe should be addressed in the Core Set revision?**

- **Yes: if yes, please describe**
- **No**

**Please describe any challenges related to the use of the Core Set in your setting and any recommendations to consider in the revision.**

Open text box

**Do you have any other suggestions for the Core Set revision.**

Open text box

**How often do you treat patients with:**

|  | **Often** | **Sometimes** | **Rarely** | **Never** |
| --- | --- | --- | --- | --- |
| Acute neurologic conditions (i.e., < 6 months since diagnosis) |  |  |  |  |
| Chronic stable neurologic conditions (i.e., >6 months since diagnosis but not expected to worsen with time) |  |  |  |  |
| Chronic progressive neurologic conditions (i.e., >6 months since diagnosis but with the potential for the condition to worsen) |  |  |  |  |

**In what clinical setting do you primarily work?**

- Acute care hospital
- Inpatient rehabilitation facility
- Skilled nursing facility
- Long-term acute care facility (e.g., LTAC)
- Long-term nursing facility (e.g., nursing home)
- Outpatient – Hospital-based clinic
- Outpatient – Private clinic
- Home health
- Other: If other, please note your clinical setting.

**Is your clinical setting a for profit or non-profit institution/organization?**

- For profit
- Non-profit

**Other than in clinical care, for what purposes does your organization use data from the Core Set? Select all that apply.**

- Marketing
- Appealing billing-related denials
- Staff evaluation/development
- Quality improvement initiatives
- Comparison of outcomes across organizations
- Reporting at team/family conferences
- Reporting to payers
- Other: Please comment

**If you use the Core Set in your clinic, are the measures standardized (i.e., staff participated in an annual standardization/competency of the measures)?**

- Yes, if YES please describe
- No
- I don’t know
- Not applicable. We don’t use the Core Set measures in our clinic

**Are there specific fields in your medical record to document the results of each of the measures below? Select all that apply.**

- Berg Balance Scale
- Functional Gait Assessment
- Activities Specific Balance Confidence Scale (ABC Scale)
- 10-Meter Walk Test
- 6-Minute Walk Test
- 5 Times Sit to Stand Test
- None of the above

**Does your facility extract Core Set data and provide reports from the electronic medical record?**

- Yes
- No
- I don’t know

**How many physical therapists and physical therapist assistants work in your clinic?**

- < 5
- 5-10
- 11-20
- > 20

**How many years have you been practicing?**

- 0-4
- 5-9
- 10-14
- 15-19
- 20 or more

**Do you have an American Board of Physical Therapy Specialists (ABPTS) certification? If so, which one(s)? Select all that apply.**

- Yes, neurology
- Yes, geriatrics
- No
- Other, please specify

**To which organizations do you belong? Select all that apply.**

- American Physical Therapy Association
- Academy of Neurologic Physical Therapy
- Other, please list organization(s)

**In which country do you live?**

- United States
- Other, please list specify

**Educator/Researcher Questions**

*As you answer the questions that follow, please respond as related to your primary position as an educator/researcher.*

**I understand the purpose, processes, and recommendations of the Core Set of Outcome Measures CPG.**

- Yes
- No

**The Core Set of Outcome Measures CPG changed my knowledge.**

- Yes, if YES HOW?
- No

**The Core Set of Outcome Measures CPG changed my work.**

- Yes, if YES HOW?
- No

**The Core Set provides a comprehensive measure of balance.**

- Strongly agree
- Agree
- Neutral
- Disagree
- Strongly disagree

**The Core Set provides a comprehensive measure of gait.**

- Strongly agree
- Agree
- Neutral
- Disagree
- Strongly disagree

**In your research focused on static and dynamic balance, do you use the Berg Balance Scale?**

- Always
- Frequently
- Seldom
- Never
- Not applicable for my research

**In your research focused on walking balance, do you use the Functional Gait Assessment?**

- Always
- Frequently
- Seldom
- Never
- Not applicable for my research

**In your research focused on balance confidence, do you use the Activities Specific Balance Confidence Scale (ABC)?**

- Always
- Frequently
- Seldom
- Never
- Not applicable for my research

**In your research focused on walking speed, do you use the 10-meter Walk Test (10mWT)?**

- Always
- Frequently
- Seldom
- Never
- Not applicable for my research

**In your research focused on walking distance, do you use the 6-minute Walk Test (6MWT)?**

- Always
- Frequently
- Seldom
- Never
- Not applicable for my research

**In your research focused on transfers, do you use the 5 Times Sit to Stand Test (5xSTS)?**

- Always
- Frequently
- Seldom
- Never
- Not applicable for my research

**In your research focused on patient goals, do you use an outcome measure such as the Goal Attainment Scale?**

- Always
- Frequently
- Seldom
- Never
- Not applicable for my research

**For each clinical utility factor below, identify its importance in selecting Core Set measures. Please indicate any other clinical utility factors that should be used as a criterion for selecting measures.**

|  | **Very Important** | **Somewhat important** | **Not important** |
| --- | --- | --- | --- |
| Time to administer and score: < 20 minutes/ measure |  |  |  |
| Cost: available at no cost |  |  |  |
| Equipment: no specialized equipment |  |  |  |

Other: please comment:

**I feel it is important to administer a measure in the Core Set even though a patient may score near 0 on the test.**

- Strongly agree
- Agree
- Neutral
- Disagree
- Strongly disagree

**In my institution, students are required to read the Core Set CPG.**

- Yes
- No
- I don’t know

**In my institution, we teach students to administer the…(select all that apply)**

- Berg Balance Scale
- Functional Gait Assessment
- Activities Specific Balance Confidence (ABC) Scale
- 10-Meter Walk Test
- 6-Minute Walk test
- 5 Times Sit to Stand Test

**In my institution, we teach students to…(select all that apply)**

- Document patient goals using an outcome measure (such as the Goal Attainment Scale)
- Discuss Core Set results with patients and use collaborative/shared decision making with patients
- Report a patient’s Core Set results in team conferences
- Score a 0 if a patient is unable to perform a Core Set measure at that time, but has goals and the capacity to do so. later
- Use standardized administration with the protocols developed by the Core Measures CPG Knowledge Translation Task Force
- 5 Times Sit to Stand Test

**In my institution, students are taught to use the Core Set …(select all that apply)**

- At initial evaluation
- At discharge
- At least once in between
- For patients with acute neurologic conditions (<6 months since diagnosis)
- For patients with chronic stable neurologic conditions (>6 months since diagnosis)
- For patients with chronic progressive neurologic conditions (>6 months since diagnosis)

**Which Academy of Neurologic Physical Therapy Education Center Modules have you (or colleagues at your institution) used in teaching your students? Select all that apply.**

- Berg Balance Scale
- Functional Gait Assessment
- 10-Meter Walk and 6-Minute Walk Test Course
- 5 Times Sit to Stand Test and Activities Specific Balance Confidence (ABC) Scale
- Score interpretation and continuum use
- None

**Are there any gaps you believe should be addressed in the Core Set revision?**

- Yes, if yes, please describe
- No

**Are there any challenges related to teaching the Core Set? Please describe any challenges and provide suggestions on how the CPG revision might address these.**

Open text box

**Do you have any other suggestions for the Core Set Revision?**

Open text box

**How many years have you been practicing?**

- 0-4
- 5-9
- 10-14
- 15-19
- 20 or more

**How many years have you been teaching?**

- 0-4
- 5-9
- 10-14
- 15-19
- 20 or more

**Do you have an American Board of Physical Therapy Specialists (ABPTS) certification? If so, which one(s)? Select all that apply.**

- Yes, neurology
- Yes, geriatrics
- No
- Other, please specify

**To which organizations do you belong? Select all that apply.**

- American Physical Therapy Association
- Academy of Neurologic Physical Therapy
- Other, please list organization(s)

**In which country do you live?**

- United States
- Other, please list specify

**Manager/Supervisor/Administrator Questions**

*As you answer the questions that follow, please respond as related to your primary position as a manager/supervisor/administrator.*

**I understand the purpose, processes, and recommendations of the Core Set of Outcome Measures CPG.**

- Yes
- No

**The Core Set of Outcome Measures CPG changed my knowledge.**

- Yes, if YES HOW?
- No

**The Core Set of Outcome Measures CPG changed my work.**

- Yes, if YES HOW?
- No

**The Core Set provides a comprehensive measure of balance.**

- Strongly agree
- Agree
- Neutral
- Disagree
- Strongly disagree

**The Core Set provides a comprehensive measure of gait.**

- Strongly agree
- Agree
- Neutral
- Disagree
- Strongly disagree

**If you use the Core Set in your clinic, are the measures standardized (i.e., staff participated in an annual standardization/competency of the measures)?**

- Yes, if YES please describe
- No
- I don’t know
- Not applicable. We don’t use the Core Set measures in our clinic

**Does your facility have a required set of outcome measures? Please select all that apply.**

- No
- Yes, including the Berg Balance Scale
- Yes, including the Functional Gait Assessment
- Yes, including the Activities Specific Balance Confidence Scale (ABC Scale)
- Yes, including the 10-Meter Walk Test
- Yes, including the 6-Minute Walk Test
- Yes, including the 5 Times Sit to Stand Test
- Other: please identify other measure(s)

**Are there specific fields in your medical record to document the results of each of the measures below? Select all that apply.**

- Berg Balance Scale
- Functional Gait Assessment
- Activities Specific Balance Confidence Scale (ABC Scale)
- 10-Meter Walk Test
- 6-Minute Walk Test
- 5 Times Sit to Stand Test
- None of the above

**Other than in clinical care, for what purposes does your organization use data from the Core Set? Select all that apply.**

- Marketing
- Appealing billing-related denials
- Staff evaluation/development
- Quality improvement initiatives
- Comparison of outcomes across organizations
- Reporting at team/family conferences
- Reporting to payers
- Other: Please comment

**For each clinical utility factor below, identify its importance in selecting Core Set measures. Please indicate any other clinical utility factors that should be used as a criterion for selecting measures.**

|  | **Very Important** | **Somewhat important** | **Not important** |
| --- | --- | --- | --- |
| Time to administer and score: < 20 minutes/ measure |  |  |  |
| Cost: available at no cost |  |  |  |
| Equipment: no specialized equipment |  |  |  |

Other: please comment:

**Does your facility extract Core Set data and provide reports from the electronic medical record?**

- Yes
- No

**Have you modified the Core Set in your setting?**

- Yes
- No

**Are there any gaps you believe should be addressed in the Core Set revision?**

- Yes, if yes, please describe
- No

**Do you have any other suggestions for the Core Set Revision?**

Open text box

**How many physical therapists and physical therapist assistants work in your clinic?**

- < 5
- 5-10
- 11-20
- > 20

**How many years have you been practicing?**

- 0-4
- 5-9
- 10-14
- 15-19
- 20 or more

**Do you have an American Board of Physical Therapy Specialists (ABPTS) certification? If so, which one(s)? Select all that apply.**

- Yes, neurology
- Yes, geriatrics
- No
- Other, please specify

**To which organizations do you belong? Select all that apply.**

- American Physical Therapy Association
- Academy of Neurologic Physical Therapy
- Other, please list organization(s)

**In which country do you live?**

- United States
- Other, please list specify

**How long have you been a Supervisor/manager/administrator?**

- 0-4
- 5-9
- 10-14
- 15-20
- More than 20

**In what clinical setting do you primarily work?**

- Acute care hospital
- Inpatient rehabilitation facility
- Skilled nursing facility
- Long-term acute care facility (e.g., LTAC)
- Long-term nursing facility (e.g., nursing home)
- Outpatient – Hospital-based clinic
- Outpatient – Private clinic
- Home health
- Other: If other, please note your clinical setting.

**Is your clinical setting a for profit or non-profit institution/organization?**

- For profit
- Non-profit
